# The Emergence of Echinocandin-Resistant *Candida glabrata* Exhibiting High MICs and Related *FKS* Mutations in Turkey

**DOI:** 10.3390/jof7090691

**Published:** 2021-08-26

**Authors:** Ali Korhan Sig, Meliha Cagla Sonmezer, Dolunay Gülmez, Serhat Duyan, Ömrüm Uzun, Sevtap Arikan-Akdagli

**Affiliations:** 1 Department of Medical Microbiology, Faculty of Medicine, Hacettepe University, 06230 Ankara, Turkey; dr_korhan@hotmail.com (A.K.S.); dolunayglm@gmail.com (D.G.); drsduyan@gmail.com (S.D.); 2 Department of Infectious Diseases and Clinical Microbiology, Faculty of Medicine, Hacettepe University, 06230 Ankara, Turkey; caglasonmezer@hotmail.com (M.C.S.); omrumuzun@gmail.com (Ö.U.)

**Keywords:** echinocandin resistance, *Candida glabrata*, candidemia

## Abstract

The frequency of invasive fungal infections shows a rising trend as well as a high morbidity and mortality. Among the causative agents, a shift toward the non-albicans *Candida* species including *Candida glabrata* species complex is being observed in several centers. Echinocandin resistance is increasingly published; however, isolates presenting with an in vitro resistance have not yet been reported from Turkey. We, herein, report the first *FKS* mutant and phenotypically echinocandin-resistant *C. glabrata* clinical strains from a single center in Turkey. In a 43-year-old female patient, several enterocutaneous fistulae developed after a long term hospitalization period and several complicated surgeries. She eventually required parenteral nutrition via a tunneled central venous catheter (CVC). Following a number of bacteremic and fungemic episodes as well as intensive antimicrobial interventions (including fluconazole, caspofungin and anidulafungin), a CVC-related candidemia caused by *C. glabrata* was detected. The isolated strain yielded high minimum inhibitory concentration (MIC) values for echinocandins and was categorized as resistant. A resistance-related mutation was detected in *FKS2* HS1 (D666V). Blood cultures remained negative after the removal of the CVC and treatment with caspofungin and high-dose fluconazole. Following this first case, two additional *C. glabrata* strains with high echinocandin MICs were isolated from the urine cultures of two unrelated patients from different wards with different mutations in *FKS2* HS1 (S663P and delF659). Our findings indicate that routine antifungal susceptibility testing is crucial and underlines the need for attention for the increasing trend of acquired echinocandin resistance in *C. glabrata*.

## 1. Introduction

Invasive fungal infections are observed more frequently due to an increased number of patients harboring major risk factors and, unfortunately, they have high mortality rates [1]. The mortality rate of candidemia can vary according to the immune status, age and comorbidities of the host. The all-cause mortality in patients can be as high as 25%, reaching 32% among cases ≥65 years of age and up to 70% in fulminant sepsis [2,3]. Although *Candida albicans* has been the most common causative agent for a long time, epidemiologic variations due to geographic locations, treatment regimens and patient conditions have emerged and a shift toward non-albicans *Candida* species has been reported [2,3,4,5]. An increasing use of antifungals as a prophylactic and preemptive treatment may be a part of this trend, giving rise to azole non-susceptible species including the *Candida glabrata* species complex (SC) [2,5,6].

*C. glabrata* SC mainly causes infections among the elderly and solid organ transplant recipients [3]. It is reported to be the second fungal cause of bloodstream infections following *C. albicans* in several geographical regions such as the United States and northwestern Europe [6]. A 10-year retrospective candidemia survey from our hospital showed that *C. glabrata* SC was the fourth most common *Candida* species [7]. A further investigation of the laboratory data revealed increasing rates of *C. glabrata* SC from 2008 to 2019, ranking as the second most common SC from all clinical specimens and third among blood isolates [4].

The diagnosis and management of invasive candidiasis (IC) have been defined in the guidelines for different patient groups. Echinocandins have different roles with a varied strength of recommendation (SoR) and quality of evidence (QoE) in distinct patient populations. For the treatment of IC in non-neutropenic patients, echinocandins are recommended as a first line therapy in different guidelines [8,9]. However, echinocandin resistance caused by *FKS1* and/or *FKS2* mutations is an emerging problem that is especially problematic for an azole non-susceptible species such as *C. glabrata* SC and is directly related to a higher mortality [3,10]. The Clinical and Laboratory Standards Institute (CLSI) and The European Committee on Antimicrobial Susceptibility Testing (EUCAST) have documented standard methods to test and evaluate antifungal susceptibility including echinocandins [11,12,13]. Echinocandin resistance in *C. glabrata* SC, defined as a determination of the minimum inhibitory concentration (MIC) values above the established breakpoints and accompanied by a demonstration of related *FKS* mutations in several studies, has been reported previously from many regions including Europe, Asia, Africa and the Americas [10,14]. Based on these, the resistance rates vary from one country/center to another and may exceed 10% [10,15]. A prior antifungal (including echinocandin) exposure was also associated with in vitro multi-drug resistance (to azoles, echinocandins and polyenes) in *C. glabrata* SC [3] and there are also reports of acquiring in vivo resistance during antifungal therapy [16,17,18]. Thus, echinocandin resistance tends to increase in *C. glabrata* SC isolates following azole or echinocandin treatments [19].

In a 12-center study from Turkey by Arikan-Akdagli et al. where 216 *C. glabrata* SC strains isolated in the time period of 1997–2017 were also tested, no in vitro echinocandin-resistant strains with MICs above the established breakpoints were detected [20]. Among the strains included in the SENTRY Antifungal Surveillance Program (2013), on the other hand, one *C. glabrata* SC strain from Turkey was reported to be resistant to anidulafungin, susceptible to micafungin and in an intermediate category for caspofungin. This isolate, however, yielded a wild type genotype in terms of *FKS1* and *FKS2* sequences with no resistance-related mutations [14]. Additionally, in one recent report, resistance-related *FKS* mutations were detected in three *C. glabrata* strains isolated from a single center in Turkey. However, these strains were in the susceptible (*n* = 1) or intermediate (*n* = 2) category when tested by the CLSI microdilution method for anidulafungin and micafungin and no in vitro echinocandin resistance could be detected [14,21]. As a result, and to our knowledge, *C. glabrata* SC strains exhibiting both phenotypic (high echinocandin MICs) and molecular resistance (*FKS* mutations) have not been previously reported from Turkey.

We herein report a case from Turkey where a *C. glabrata* strain determined to be resistant to echinocandins in vitro and harboring resistance-related FKS mutations was isolated from a blood culture. We also provide information regarding two more echinocandin-resistant *C. glabrata* strains in our center that followed the isolation of this very first case.

## 2. Case Report

The first patient was a 43-year-old woman with a lengthy and complicated hospital course. She was admitted to our surgical department with symptoms of abdominal pain, nausea and vomiting for the previous two months. Her medical history was significant for a choledochal cyst operation 34 years ago; a Roux-en-Y hepaticojejunostomy after a post-cholecystectomy 27 years ago and finally a left salpingectomy plus a lower intestinal resection and end-to-end anastomosis that was performed 16 months prior to admission. Since that last operation, she had undergone several abdominal surgeries to control a biliary leakage. Eventually, she developed several enterocutaneous fistulae. The patient was on parenteral nutrition via a tunneled central venous catheter (CVC; Hickman catheter). In the preceding five months after admission to the hospital, episodes of bacteremia caused by multi-drug-resistant *Acinetobacter baumannii*, carbapenem-resistant *Klebsiella pneumoniae*, *Morganella morganii*, *Enterococcus faecium*, *C. albicans* and *C. glabrata* occurred. She received broad-spectrum antibiotic treatments (ertapenem, meropenem, colistin and teicoplanin) and antifungal drugs (fluconazole, caspofungin and anidulafungin) for multiple episodes of bloodstream infections.

Five months after admission to the surgical ward, the body temperature of the patient rose to 39.5 °C and leukocytosis (13,200 cells/µL) and high C-reactive protein levels (15.3 mg/dL) were detected. She had no symptoms potentially related to respiratory or urinary tract infections. A chest radiograph showed no pneumonia patch and the urinalysis was normal. Blood cultures from the CVC and peripheral vein were detected as positive for yeasts. The tunneled CVC was considered to be a possible infection source and therefore removed; the catheter, catheter blood and venous blood cultures were examined. Echinocandin-resistant *C. glabrata* and *C. albicans* were isolated from the blood culture in the fifth month after hospitalization (day 0). Caspofungin was administered (70 mg loading dose followed by 50 mg/day) when the yeast growth signal was reported on day −3 of the blood cultures until day 18. The antifungal treatment was not revised as multiple follow-up blood cultures remained sterile after the CVC removal with the clearance of the infecting *C. glabrata* and *C. albicans* strains at day 5.

The patient had a protracted and complicated hospitalization and died one year after admission due to a cerebral air embolization. This last incident of embolization was previously reported in a visual case presentation [22].

The patient data were extracted from the hospital records retrospectively. The study was conducted according to the guidelines of the Declaration of Helsinki and was approved by the Clinical Research Ethics Committee of Hacettepe University (No.: GO-21/518).

## 3. Other Echinocandin-Resistant *C. glabrata* Strains Isolated in Our Center Following This First Case

More than a year after the isolation of the echinocandin-resistant strain from this first case, two additional *C. glabrata* strains (Strains No. 2 and 3; Table 1) with high micafungin and anidulafungin MICs were isolated from the urine cultures of two patients who were hospitalized in different wards located in different hospital buildings in our center. No other clinical specimens of these two patients yielded a growth of *C. glabrata* and the patients were clinically evaluated as colonized.

## 4. Isolation and Identification of the Strains, Detection of In Vitro Echinocandin Resistance and Molecular Analysis

The strains were isolated from blood or urine cultures and the methods followed for the isolation, identification, in vitro echinocandin susceptibility and detection of molecular resistance are overviewed below.

BACTEC Plus Aerobic/F bottles (Becton Dickinson, Franklin Lakes, NJ, USA) were used for the blood cultures. Positive culture bottles were examined by Gram staining and in the case of the detection of yeast cells in direct microscopy, subcultures onto Sabouraud Dextrose agar (SDA, Acumedia, Lancashire, UK) and *Candida* chromogenic agar (HiMedia Laboratories, Mumbai, India) were performed in addition to routine bacteriological subcultures onto sheep blood agar (RTA Laboratories, Kocaeli, Turkey), chocolate agar (RTA Laboratories, Kocaeli, Turkey) and Eosin Methylene Blue (EMB) agar (RTA Laboratories, Kocaeli, Turkey). 

Urine specimens were subcultured onto blood and EMB agar media and the culture plates were sent to the Mycology Laboratory in case yeast growth was detected. 

A germ tube test was performed for all isolates. The strains were initially identified using ID32C (bioMérieux, Marcy-l’Étoile, France) and a morphologic examination on corn meal Tween 80 agar [23]. The identification was re-evaluated with MALDI-TOF MS (Bruker, Billerica, MA, USA) yielding scores of ≥2 in a duplicate analysis and finally confirmed as *C. glabrata* sensu stricto by the sequencing of the *ITS* gene [24]. 

In vitro antifungal susceptibility testing (AFST) of the isolates was done by broth microdilution according to both EUCAST and CLSI recommendations and the MIC values were evaluated using the clinical breakpoints and epidemiological cut-off values of the testing method used [11,12,13,25]. 

The molecular mechanisms of echinocandin resistance were investigated via the sequencing of the *FKS1* and *FKS2* genes [18].

## 5. Results and Discussion

The in vitro susceptibility test results are depicted in Table 1. AFST by both EUCAST and CLSI reference microdilution methods revealed high MICs and a resistant category for micafungin and anidulafungin for all strains and yielded the same results upon repetition (Table 1). As recommended, micafungin and anidulafungin MICs were determined to explore the in vitro resistance to echinocandins [11,13,26]. 

The sequencing of the *FKS1* and *FKS2* genes revealed a D666V mutation in the *FKS2* hot spot 1 (HS1) region in the strain isolated from the reported case (Table 1). This mutation was previously documented in echinocandin-resistant *C. glabrata* isolates with additional *FKS1* HS1 mutations [10,27]. We did not detect any mutations in *FKS1* or additional mutations in *FKS2* for this strain. The consecutive echinocandin-resistant *C. glabrata* isolates, Strains No. 2 and 3 (Table 1), had mutations in *FKS2* HS1. Strain No. 2 had a S663P mutation and Strain No. 3 had a deletion at F659 (Table 1). Both mutations were reported from echinocandin-non-susceptible *C. glabrata* isolates previously reported [10,14,21,27,28].

As a limitation, the investigations for the genotyping of the strains were not included in this study. All three strains were obtained from patients from different wards in different hospital buildings. Strains No. 2 and 3 were detected more than a year later and no other echinocandin-resistant *C. glabrata* strains have been detected to date during routine antifungal susceptibility testing, which is performed for all clinical *C. glabrata* isolates in our center. In addition, the three resistant strains harbored different echinocandin resistance-related mutations. These findings suggest that clonal spread does not appear likely although it is not totally ruled out.

In the 2019 AR threat report, the CDC declared the drug-resistant *Candida* species as a serious threat [29]. Although echinocandins are effective for most *Candida* species, an acquired resistance has been reported and is increasing worldwide [10]. This short communication article is the first report from Turkey for *C. glabrata* strains that exhibit both a phenotypic and molecular resistance to echinocandins. The phenotypic resistance was detected by both EUCAST and CLSI reference methods. Our findings underline the need for attention for this growing problem in *C. glabrata* strains with respect to secondary echinocandin resistance.

## Figures and Tables

**Table 1:** The results of the antifungal susceptibility testing with the EUCAST and CLSI reference methods and *FKS* mutations for the echinocandin-resistant *Candida glabrata* isolates reported in this article.

Strain No.	Antifungal	EUCAST (mg/L)				CLSI (mg/L)				Mutation *FKS2* HS1
		MIC	ECOFF	CBP	Interpretation	MIC	ECOFF	CBP	Interpretation	
1 *	Amphotericin B	0.5	1	1	S	1	NA			D666V
	Fluconazole	32	32	0.002	SDD	8	32	-	SDD	
	Voriconazole	1	1	NA	WT	0.25	0.5	NA	WT	
	Anidulafungin	0.5	0.064	0.064	R	0.5	0.25	0.125	R	
	Micafungin	0.125	0.032	0.032	R	0.25	0.032	0.064	R	
2	Amphotericin B	0.125	1	1	S	0.25	NA			S663P
	Fluconazole	2	32	0.002	SDD	4	32	-	SDD	
	Voriconazole	0.06	1	NA	WT	0.06	0.5	NA	WT	
	Anidulafungin	2	0.064	0.064	R	4	0.25	0.125	R	
	Micafungin	4	0.032	0.032	R	2	0.032	0.064	R	
3	Amphotericin B	0.25	1	1	S	0.5	NA			delF659
	Fluconazole	2	32	0.002	SDD	4	32	-	SDD	
	Voriconazole	0.06	1	NA	WT	0.06	0.5	NA	WT	
	Anidulafungin	1	0.064	0.064	R	4	0.25	0.125	R	
	Micafungin	4	0.032	0.032	R	2	0.032	0.064	R	

ECOFF: epidemiologic cut-off value; CBP: clinical breakpoint; NA: not applicable/not applied; S: susceptible; R: resistant; SDD: susceptible dose-dependent; WT: wild type. * Strain No. 1 was isolated from the blood culture of the patient reported in detail in this article.
